# Functional Recovery of Cranial Nerves in Patients with Traumatic Orbital Apex Syndrome

**DOI:** 10.1155/2017/8640908

**Published:** 2017-11-13

**Authors:** Zhenxing Li, Danfeng Zhang, Jigang Chen, Junyu Wang, Liquan Lv, Lijun Hou

**Affiliations:** Department of Neurosurgery, Shanghai Institute of Neurosurgery, Shanghai Changzheng Hospital, Second Military Medical University, Shanghai 200003, China

## Abstract

**Objective:**

Traumatic orbital apex syndrome (TOAS) is a rare disease characterized by the damage of cranial nerves (CNs) II, III, IV, and VI. The aim of our study was to analyze the functional recovery of CNs in TOAS and discuss the management of these patients.

**Methods:**

We retrospectively reviewed 28 patients with TOAS treated in the Department of Neurosurgery, Shanghai Changzheng Hospital from February 2006 to February 2016. Functional recovery of CNs was evaluated based on extraocular muscle movement and visual perception. Follow-up duration was at least 6 months.

**Results:**

There were 26 males and 2 females with a mean age of 35.3 years. The most common cause of TOAS was traffic accident. CN IV suffered the lightest injury among CNs III, IV, and VI. CN II achieved obvious improvement at 3-month follow-up, while other CNs enjoyed evident improvement at 6-month follow-up. There was no significant difference between conservative treatment and surgical decompression.

**Conclusion:**

CNs passing through orbital apex region might recover to different degrees several months after proper management. Clinical decision should be individualized and surgical decompression could be considered with evidence of fracture, hematoma, or deformation.

## 1. Introduction

Orbital apex syndrome (OAS) is a rare and complex disease where the visual damage combines with the superior orbital fissure syndrome (SOF) [1]. It is characterized by ophthalmoplegia, ptosis, and visual loss due to the damage of cranial nerves (CNs) II, III, IV, and VI [2–5]. OAS may be caused by trauma, infection, neoplasm, inflammation, and vascular disease, among which trauma is one of the most common causes [1]. However, there are few literatures regarding the diagnosis and treatment of traumatic OAS (TOAS). What is more, guidelines for the management of TOAS were unavailable based on scattered cases owing to the low incidence of TOAS and lack of prospective or controlled studies [6]. Current treatment of TOAS commonly counts on the experiences of different institutions with variable conclusions. Though surgical decompression was suggested by some studies, several other authors proved that simple mega dose corticosteroid treatment or follow-up without management might be effective as well [7].

In present study, we, respectively, summarized the treatment experience of 28 cases diagnosed with TOAS. The primary goal was to analyze clinical features, imaging findings, and treatment modes of TOAS as well as prognosis of these patients.

## 2. Materials and Methods

### 2.1. Patients

From February 2006 to February 2016, 28 cases of TOAS were identified from 1802 traumatic brain injury patients treated in the Department of Neurosurgery, Shanghai Changzheng Hospital. Cases resulting from inflammatory, infectious, neoplastic, and vascular conditions were excluded from this study. Diagnosis of TOAS was made based on traumatic history, symptoms, physical examinations, and imaging findings. Every case underwent detailed ophthalmologic examination and they all had complete or incomplete symptoms of impaired vision, ophthalmoplegia, fixed and dilated pupil, and disappearing direct and indirect light reflex. Two grading scales were proposed to evaluate the severity and recovery of CN injury. For CN II, visual perception was assorted into four levels: 0, no light perception; 1, light perception; 2, hand move; 3, finger counting or better [8]. For CNs III, IV, and VI, functional scoring was based on the extraocular muscle movement: 0, complete fixed eyeball and no movement; 1, minor movement; 2, obvious movement; 3, complete movement [9]. To analyze the neurologic recovery status of each CN, the recovery degree was defined as the result of that score at one later time point minus the score at a previous time point.

*Treatment*. Among 28 cases of TOAS, 8 of them underwent conservative treatment including intravenous drip of methylprednisolone (500–1000 mg/day for 2-3 days) and vasodilators. Mecobalamin and vitamin B_1_ were also injected to nourish the CNs. Twenty cases were included in the surgical treatment and the indications were as follows: (1) the duration from injury to admission of less than 1 week; (2) patients with abrupt disturbance of vision or eye movements; (3) patients with obvious imaging evidence of fracture, hematoma, or deformation in orbital apex region, or patients without abnormality in orbital apex region but conservative treatment (methylprednisolone) for 72 hours proving to be ineffective. For these 20 patients 9 with obvious facture in the optic canal or sharp decline of vision received surgical decompression of the optic canal. Three cases with evidence of SOF compression on CT scans underwent surgical decompression of SOF. Eight cases received optic canal and SOF decompression at the same time due to the abnormal changes in both the optic canal and SOF. All these operations were conducted via the transcranial approach, and cortical steroids, neurotrophic agents, were administrated for all patients after surgery. In sum, 18 and 10 patients underwent optic nerve canal decompression (ONCD) and non-ONCD treatment for CN II injury, respectively. Likewise, 11 and 17 patients underwent superior orbital fissure decompression (SOFD) and non-SOFD treatment for CNs III, IV, and VI injury, respectively. The patients were followed up by out-patient review and telephone, and the duration was at least 6 months.

### 2.2. Statistical Analysis

Continuous data were reported as mean ± standard deviation (SD). The Wilcoxon signed rank test was used for the paired data and the Kruskal-Wallis test was used for multiple independent groups. *P* < 0.05 was considered statistically significant.

## 3. Results

### 3.1. Demographics and Clinical Presentations

92.9% (26/28) of the cases were male. The mean age was 35.3 with a range of 9 to 62 years old. The most common injury mechanism was traffic accident (18/28), followed by tumbling (7/28) and hit by falling object (3/28). All patients were complicated with orbit fracture and zygomatic fracture was found in 7 patients. Careful physical examinations were performed upon admission and all patients presented with complete or incomplete visual loss. According to the grading scale, mean score for CN II was 0.54 ± 0.74 (Table 1). Different degrees of confined extraocular muscle movement with injuries in CNs III, IV, and VI were also observed. Mean score for CNs III, IV, and VI was 0.68 ± 0.72, 1.18 ± 0.77, and 0.54 ± 0.69, respectively. Significant differences in grading scores were detected among CNs III, IV, and VI using Kruskal-Wallis test (Table 1). The Bonferroni correction was used to compare the functional scores of either two cranial nerves of extraocular muscle. Statistically significant differences were found between CNs IV and III (*P* = 0.018) and between CNs IV and VI (*P* = 0.003), indicating that CN IV suffered the lightest injury in TOAS.

### 3.2. CN Recovery

Only 4 cases (14.3%) had improved vision 3 months after the injury and mean score of CN II was 0.68 ± 0.82. At 6 months, 5 patients (17.9%) achieved improved vision with a mean score of 0.71 ± 0.85 (Table 1). Noticeably, no patient had a full recovery for optic nerve function after trauma. Significant improvement was spotted between 0 and 3 months for CN II (*P* = 0.046) and the recovery degree was 0.14 ± 0.36 (Table 2). At 3 months, 23 patients (82.1%) regained functional improvement for at least one CN responsible for extraocular muscle movement. This number increased to 26 (92.9%) at 6 months. The mean scores for CNs III, IV, and VI were 1.14 ± 0.71, 1.43 ± 0.69, and 1.35 ± 0.91 at 3 months, respectively. There was no significant difference between these 3 CNs (*P* = 0.325). As for function status at 6 months, CN IV had the highest score (1.96 ± 0.79), whereas CN III had the lowest score (1.75 ± 0.84). Score for CN VI was 1.86 ± 0.88 with no significant differences between these 3 CNs (*P* = 0.640) (Table 2). All 3 CNs presented significant improvement from 0 to 3 months as well as from 3 to 6 months (*P* < 0.01). Moreover, CN VI showed the greatest recovery degree (0.82 ± 0.82) during the first 3 months and CN III showed more obvious recovery degree (0.61 ± 0.74) compared to other two CNs during the second 3 months (Table 2).

### 3.3. Recovery of CN with ONCD and SOFD

In our cases, 18 cases underwent ONCD treatment and 10 patients underwent non-ONCD treatment for their CN II injury. There were no statistically significant differences in functional scores between two groups at initial injury, 3 and 6 months. Similarly, 11 patients received SOFD treatment and 17 received non-SOFD treatment for their CNs III, IV, and VI injury. No statistically significant differences for functional scores of the 3 CNs were noticed between two groups at initial injury, 3 and 6 months (Table 3).

### 3.4. Illustrative Case

**Figure 1:**
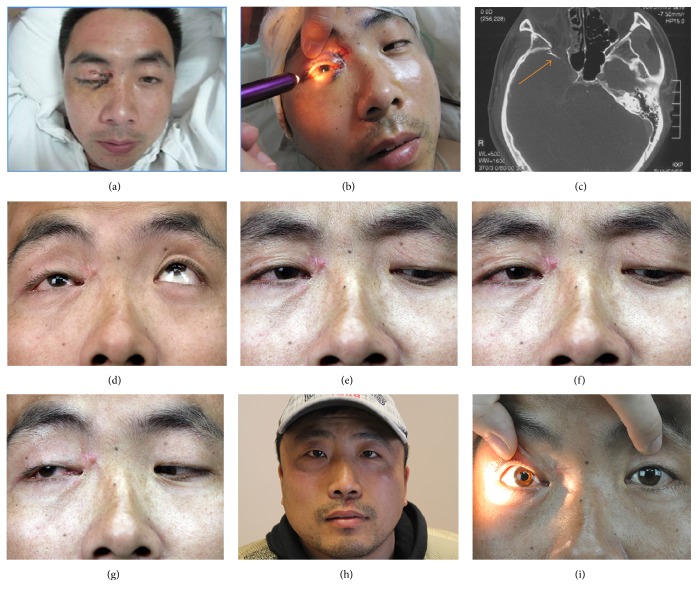
(a) Preoperative physical examination showed ptosis of right eye and contusion of the surrounding soft tissues; (b) dilated pupil of the right eye before surgery; (c) presurgical CT demonstrated fractures of right orbital apex (the orange arrow means there were obvious fractures in the orbital apex region); (d–g) the eye movement was significantly improved with slightly restriction at 3 months after the operation.

A 32-year-old male was admitted to our department complaining from blindness and ptosis in right eye 3 days after injury. He was conscious and physical examination on admission suggested ptosis, dilated pupil, ophthalmoplegia, no light perception, and disappearing direct and indirect light reflex in right eye (Figures 1(a) and 1(b)). Head CT scanning indicated optic nerve compression and fractures in the orbital apex region (Figure 1(c)). The patient received surgical decompression of optic nerve and SOF after 3 days of mega dose corticosteroid therapy. Neurotrophic agents were administrated after operation. The postoperative course was uncomplicated. The symptoms got significant improvement while eye movements were slightly restricted at 3 months after the surgery (Figures 1(d)–1(g)). Follow-up results suggested the patient got complete CN recovery at 6-month follow-up (Figures 1(h) and 1(i)).

## 4. Discussion

TOAS is a rare complication of craniomaxillofacial trauma, characterized by traumatic optic neuropathy (TON) combining with the SOF syndrome [1]. There is no accurate data about its incidence according to the literature review [10]. In our department, only 28 cases of TOAS were identified and the incidence in our single center is 1.55% (28/1802). Diagnosis of TOAS usually depends on the clinical presentations. However, it should be noticed that unconscious patients might be ignored. In our study, 3 cases fell into a coma after injury and when they got awake, TOAS was confirmed rightly based on their complaints and physical examinations. Thus, unconscious patients with traumatic zygomatic or maxillary facture should be carefully examined for potential TOAS in case of missing optimal therapeutic opportunity.

The orbital apex region is a narrow and complex anatomical area with various neurovascular structures passing through [11]. Therefore, even minor craniofacial force might cause neurological disorders around the cranial apex region and thus lead to TOAS. According to degree of compression on imaging data, TOAS can be divided into two types: (1) direct damage resulting from fracture fragment, foreign bodies, or hematoma and (2) indirect damage resulting from secondary inflammation and edema [12]. It is widely acknowledged that hemorrhage inside the meningeal sheath, optic nerve swelling, and necrosis secondary to decreased blood flow could be the main pathology of TON.

Our findings confirmed that CN IV suffered from the lightest injury in TOAS among CNs III, IV, and VI (Table 1), which might owe to its special anatomical features. CN IV is the thinnest CN and locates above CN III in SOF. Compared to CNs III and VI within the common tendinous ring, CN IV travels right above the ring and might endure less traction during trauma. In our cases, CN II was severely injured with most of patients having no or slight light perception (Table 1). In the optic canal, dural sheath of CN II is closely attached to surrounding bones [13] and external impact forces can easily transmit to CN II. Moreover, CN II is very sensitive to shock pressure and hypoxia [14] and thus both primary fractures and secondary ischemia or edema can eventually cause severe optic neuropathy.

According to our results, despite the significant improvement during the first 3 months for CN II, the final functional outcomes of this nerve were not satisfactory. This might be due to the fact that the initial functional status of CN II was poor and that retinal ganglion cell was sensitive to ischemia and hypoxia which made regeneration of CN II extremely difficult. Except for CN II, all other CNs showed evident improvement and relatively satisfactory outcomes at the 6-month follow-up (Table 2). This suggested that the CNs with TOAS could achieve significant improvement with proper medical or surgical intervention. Although pleasing visual recovery was inaccessible, functional improvements of other CNs could relieve tormenting symptoms of ophthalmoplegia and facial disability.

At the 6-month follow-up, improved recovery of CN II was achieved in 5 cases (17.9%), compared with improved recovery of CNs III, IV, and VI in 26 cases (92.9%). Better recovery was indicated for CNs III, IV, and VI compared to CN II. According to a literature review of patients with TON [15], the effective rate of decompression varies from 27% to 82%, which was 40% to 60% for conservative treatment. The functional recovery of CN II in our patients with TOAS might be worse than in patients with simple TON, which seemed to be associated with different degree of damage to optic nerve.

Treatment of TOAS with corticosteroid or surgical decompression remains controversial due to its low incidence and unavailability of professional guidelines. In a report by Acartürk et al. [16], 5 cases of TOAS without obvious fracture recovered well after mega-dose corticosteroid. On the contrary, Pletcher et al. [17] proposed that simple steroid treatment might have worse prognosis compared with surgical decompression. Imaizumi et al. [12] reported that one case receiving decompression of optic canal and SOF regained visual perception successfully. While in another study [10], 2 cases of TOAS underwent conservative treatment of steroid and vitamin B12 with one of them presenting no improvement and the other one presenting incomplete eye movement. In our study, the function of CNs partially recovered with conservative treatments. Injured nerves might recover well in case of immediate administration of steroids before irreversible ischemic damage [10].

When comparing the surgical decompression with nonsurgical treatment, no statistical significant differences were found between these two groups for their functional outcomes (Table 3). Previous study concerning treatment of TON also showed that the effects of surgical decompression were doubtful [15]. And it should be noticed that optic nerve had possibility of spontaneous recovery [18]. In this way, we recommend individualized treatment for different types of TOAS. Based on our clinical experience, surgery should be considered for patients with obvious evidence of compression such as fracture, hematoma, or deformation, especially when mega dose steroid was ineffective or contraindicated. It is worth mentioning that 3D reconstructed craniofacial CT is an effective tool to detect the fractures, deformation, and other abnormal changes. According to the imaging findings, patients with no obvious changes in the optic canal and SOF can receive mainly mega dose corticosteroid treatment. Operation should be performed as early as possible to remove compression and improve microcirculation [19].

The prognosis of CNs in patients with TOAS might be affected by some factors, such as age, clinical preference, and treatment timing. Carta et al. [20] reported that age related axonal lipoperoxidation and membrane hydrolysis might lead to different outcomes. In our study, 5 patients (17.9%) achieved improved visual acuity at 6-month follow-up and their ages were all under 35 years old, which suggested younger patients might possibly have better prognosis after positive treatment than the olds. Besides, personal preference in clinical decision would affect the outcome. Moreover, there was no consensus on treatment timing and it could be another confounder. Some studies recommended mega dose steroid at 24 to 72 hours after injury followed by surgical decompression [21]. We recommend immediate treatment to avoid irreversible outcome.

Shortcomings of this study should be noticed. First, it is a retrospective study with existing flaws such as recall bias. Second, differences in initial injury severity and different time window of treatment might be confounders of our study. According to our acknowledgement, this is currently the largest case study regarding the management of TOAS, while definite conclusions are not available due to the limited cases. In this way, every case should be valued and multicenter study is needed to establish optimal treatment strategies.

## 5. Summary

TOAS is a rare complication of craniomaxillofacial injury. Injured CNs can achieve different degree of recovery after proper management, especially for CNs responsible for extraocular muscle movement. According to our decade-long clinical experiences, operation was recommended for patients with evidence of fracture, hematoma, or deformation.

## Figures and Tables

**Table 1:** Functional score of each cranial nerve at different time point.

	Optic nerve	Cranial nerve of extraocular muscle			*P*
	CN II	CN III	CN IV	CN VI	
Initial	0.54 ± 0.74	0.68 ± 0.72	1.18 ± 0.77	0.54 ± 0.69	0.005
3 months	0.68 ± 0.82	1.14 ± 0.71	1.43 ± 0.69	1.36 ± 0.91	0.325
6 months	0.71 ± 0.85	1.75 ± 0.84	1.96 ± 0.79	1.86 ± 0.89	0.640

*Note*. Calculated by using the Kruskal-Wallis test.

**Table 2:** Comparison of cranial nerve recovery rates between different time point.

Cranial nerve	Time point (month)	Mean ± SD	*P*
CN II	0–3	0.14 ± 0.36	0.046
	3–6	0.04 ± 0.19	0.317
CN III	0–3	0.46 ± 0.51	<0.001
	3–6	0.61 ± 0.74	=0.001
CN IV	0–3	0.25 ± 0.44	0.008
	3–6	0.54 ± 0.58	<0.001
CN VI	0–3	0.82 ± 0.82	<0.001
	3–6	0.50 ± 0.69	0.002

*Note*. Calculated by using Wilcoxon Signed rank test.

**Table 3:** Recovery of cranial nerve with ONCD and SOFD.

		Initial	3 months	6 months
CN II	ONCD	0.33 ± 0.49	0.44 ± 0.62	0.50 ± 0.71
	Non-ONCD	0.90 ± 0.99	1.1 ± 0.99	1.1 ± 0.99
	*P*	0.137	0.076	0.107
CN III	SOFD	0.64 ± 0.81	1.09 ± 0.83	2.00 ± 0.89
	Non-SOFD	0.71 ± 0.69	1.18 ± 0.64	1.59 ± 0.80
	*P*	0.700	0.817	0.151
CN IV	SOFD	1.09 ± 0.70	1.45 ± 0.69	1.91 ± 0.83
	Non-SOFD	1.23 ± 0.83	1.41 ± 0.71	2.00 ± 0.79
	*P*	0.562	0.895	0.665
CN VI	SOFD	0.55 ± 0.69	1.00 ± 0.89	1.91 ± 0.94
	Non-SOFD	0.53 ± 0.72	1.59 ± 0.87	1.82 ± 0.88
	*P*	0.894	0.070	0.940

*Note.* Calculated by using Wilcoxon Signed rank test.
